# Female *Salix viminalis* are more severely infected by *Melampsora* spp. but neither sex experiences associational effects

**DOI:** 10.1002/ece3.1923

**Published:** 2016-01-25

**Authors:** Kim K. Moritz, Christer Björkman, Amy L. Parachnowitsch, Johan A. Stenberg

**Affiliations:** ^1^ Department of EcologySwedish University of Agricultural SciencesP.O. Box 7044SE‐750 07UppsalaSweden; ^2^ Plant Ecology and EvolutionDepartment of Ecology and GeneticsEvolutionary Biology CentreUppsala UniversityNorbyvägen 18 DSE‐752 36UppsalaSweden; ^3^ Department of Plant Protection BiologySwedish University of Agricultural SciencesP.O. Box 102SE‐230 53AlnarpSweden

**Keywords:** Dioecy, genotypic effects, neighborhood effects, plant pathogens, sex‐biases

## Abstract

Associational effects of plant genotype or species on plant biotic interactions are common, not least for disease spread, but associational effects of plant sex on interactions have largely been ignored. Sex in dioecious plants can affect biotic interactions with herbivores and pollinators; however, its effects on plant–pathogen interactions are understudied and associational effects are unknown. In a replicated field experiment, we assessed *Melampsora* spp. leaf rust infection in monosexual and mixed sex plots of dioecious *Salix viminalis* L. to determine whether plant sex has either direct or associational effects on infection severity. We found no differences in *Melampsora* spp. infection severity among sexual monocultures and mixtures in our field experiment. However, female plants were overall more severely infected. In addition, we surveyed previous studies of infection in *S. viminalis* clones and reevaluated the studies after we assigned sex to the clones. We found that females were generally more severely infected, as in our field study. Similarly, in a survey of studies on sex‐biased infection in dioecious plants, we found more female‐biased infections in plant–pathogen pairs. We conclude that there was no evidence for associational plant sex effects of neighboring conspecifics for either females or males on infection severity. Instead, plant sex effects on infection act at an individual plant level. Our findings also suggest that female plants may in general be more severely affected by fungal pathogens than males.

## Introduction

Neighborhood composition of plant genotypes or species can affect interactions between host plants and consumers (“Associational effects”, Underwood et al. 2014). Thus far studies of associational effects have mainly focused on species, genetic or genotypic diversity or composition. Ecological plant interactions such as pathogen infection (e.g., Zhu et al. 2000; Sapoukhina et al. 2013), herbivory (e.g., Parker et al. 2010; Hambäck et al. 2014), and pollination (Genung et al. 2010) can be subject to associational effects from neighboring plants. These associational effects can be due to plant species (e.g., Parachnowitsch et al. 2014), genotypic (e.g., Parker et al. 2010) and genetic (e.g., Zhu et al. 2000) diversity. Furthermore, these interactions can drive evolutionary processes; for example, plant neighborhood effects can alter selection on plant traits mediated by biotic interactions (Parachnowitsch et al. 2014). Still, little is known about associational effects resulting from other types of variation in plants. For example, dioecious plants, which make up 5–6% of all described angiosperm species (Renner 2014), are often involved in sex‐biased biotic interactions, but effects from sex ratios of neighboring plants (associational sex effects) have not been studied. Sex effects on interactions with herbivores (reviewed by Ågren et al. 1999; Cornelissen and Stiling 2005) and pollinators (e.g., Klinkhamer and de Jong 1990; Vaughton and Ramsey 1998; Ashman 2000) of individual plants are well described across several genera, and a number of studies have also found plant sex‐biases in interactions with fungal pathogens (e.g., Åhman 1998; Chandra and Huff 2014). Because these ecological interactions can be subject to associational effects, associational plant sex effects may play an important role in plant interactions.

Pathogen spread in plants might be particularly vulnerable to associational effects. Variation in resistance genes reduces the likelihood of neighboring plants being susceptible to the same pathotype as an infected plant (Browning and Frey 1969; Leonard 1969), and greater genotypic variation in plant neighborhoods generally decreases disease susceptibility. For example, studies comparing mixtures of crop cultivars (e.g., Zhu et al. 2000; Sapoukhina et al. 2013) and of clones (Begley et al. 2009) to monocultures have found that mixtures are less susceptible to, or less severely affected by, disease. However, effects of other types of diversity have been poorly studied. Natural populations of dioecious plants often show biased abundances of the sexes (Barrett et al. 2010), and commercial plantations of dioecious species (*e.g., Salix* spp.*;* Reddersen 2001) are often monoclonal and thus monosexual. Additionally, entire stands of clonally reproducing dioecious plants can consist of single, or a few, individuals and are therefore often sex‐biased (e.g., Salicaceae, see Alliende and Harper 1989; Barrett et al. 2010). The sex ratios of neighboring plants in stands of dioecious species can be expected to have consequences for ecological interactions such as fungal pathogen infections, because genetic variation can influence infection and sex is mostly genetically determined. Furthermore, sex‐biased fungal infections are common (e.g., Alexander 1989; Quinn 1991). Currently, there is no established explanation of the mechanisms driving plant sex‐biases in fungal pathogen infections. Life history trade‐offs suggest that females would be better defended against antagonists (Rolff 2002; Vega‐Frutis et al. 2013), but studies on plant sex‐fungal disease relationships in dioecious plants include more examples of female‐biased infections than male‐biased infections (e.g., Lovett Doust and Cavers 1982; Ward 2007). Mixtures of male and female plants, the settings in which most previous field studies have been conducted, likely experience a lower severity of population infection compared to that in sexual monocultures because of higher levels of genetic variation. In addition, previous studies of plant sex effects on plant–pathogen interactions have focused solely on the sex of individual plants, ignoring the possibility of associational plant sex effects. Disregarding associational sex effects could result in effects of focal plants’ sex on fungal infection being either masked or overestimated.

**Figure 1:**
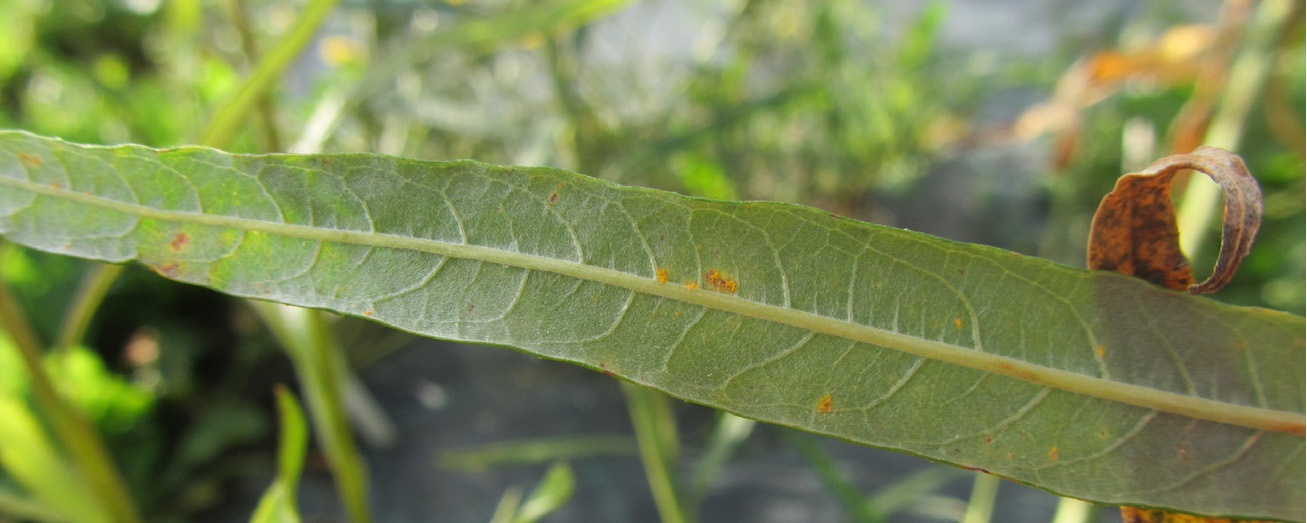
Photograph of a *Salix viminalis* leaf infected by *Melampsora* spp. Photo credit: Kim K. Moritz.

Of the dioecious plant species in which fungal plant pathogens have been studied, Salicaceae spp. infected by *Melampsora* spp. leaf rusts are among the best‐documented (e.g., Lascoux et al. 1996; Mccracken et al. 2000; Pei et al. 2004), and one study on *Salix viminalis* L. clones examined plant sex effects (Åhman 1997). In one of 2 years, female plants were more heavily infected by *Melampsora* spp. than males in an experimental mixed plantation. In this study, we compared severity of *Melampsora* spp. infection on male and female *S. viminalis* clones (Fig. 1) in field plots comprised of pure male, pure female, and mixed sex sets of genotypes to assess whether there was a sex‐biased infection rate and whether this depended on associational effects of neighborhood sex ratios. We also surveyed published studies on clone differences in infections among *S. viminalis* clones and reinterpreted the results by adding information on the clones’ sexes. Based on the findings of previous study of the plant–pathogen species pair, we hypothesized (1) that female *S. viminalis* are more susceptible to *Melampsora* spp. infection than males. If associational plant sex effects are important, we hypothesized (2) that a mix of female and male *S. viminalis* would decrease the severity of *Melampsora* spp. infection for both males and females. Because our experimental design included replicate clones of the same genotypes across monosexual and mixed sex plots we also tested (3) whether there were interactive effects of genotype and neighborhood sex ratio on the severity of infection to investigate whether clones respond differently to neighborhood sex ratios. Furthermore, we performed a literature survey to determine whether either plant sex‐bias in fungal infection of dioecious plants is more common.

## Materials and Methods

### Study species

*Salix viminalis* is naturalized in Sweden (Hylander 1971) and is the most commonly grown willow species in European Short Rotation Coppice (SRC) plantations (Pei et al. 2008). It is dioecious, and clones of both sexes are used in SRC. In our experiment, we used clones (Appendix S1) that were originally collected from Swedish naturalized populations between the late 1970s and the early 1990s for the purposes of crop research and have since then been growing in experimental gardens. Of the diseases that affect *Salix* spp., *Melampsora* leaf rusts are the most common (Pei and McCracken 2005) and serious (Ramstedt 1999), causing decreases in biomass production amounting to up to 40% in SRCs (Parker et al.^1993^; as reported in Pei et al. 2004). *Melampsora* spp. reproduce asexually multiple times over summer, and infections are easily recognized by the presence of uredia on leaves of infected plants.

### Field experiment

To capture the range in susceptibility to *Melampsora* spp. that is representative for *S. viminalis* in Sweden and to minimize the risk of single clones biasing results, we used 40 *S. viminalis* clones in our study. Commercial plantations often use only one to a few clones. Cuttings of 20 clones per sex were collected in March 2013 from experimental common gardens south of Uppsala, Sweden (Latitude: 59°80, Longitude: 17°66) and stored at −5°C for approximately 2 months. In May, we planted cuttings in 30 experimental plots east of Uppsala, Sweden (Latitude: 58°83, Longitude: 17°78). The study area is an open agricultural landscape, and plots were adjacent to fields of *Hordeum vulgare*,* Brassica napus,* and *Trifolium* spp.; nearby tree species include potential *Melampsora* spp. hosts such as willows (mainly *S. caprea* and *S. cinerea*) and larch (*Larix decidua*). Each plot comprised 40 cuttings, two cuttings per clone, and belonged to one of three treatments: monosexual male, monosexual female, or mixed sex plot. To avoid overestimating effects of sex, sex diversity or genotype because of spatial scale (see Tack et al. 2012), we used the same 40 clones in the whole experiment and arranged the plots into 10 blocks. We were able to account for sex‐ and clone‐dependent variation both within and among plots because the number of genotypes was equal among plots. Mixed sex plots contained 10 clones of each sex, distributed randomly over plots so that each clone was present in an equal number of plots. Monosexual plots contained either 20 male or 20 female clones. Plots were 10·10 m with *S. viminalis* planted in centrally placed grids. We left 70 cm between cuttings, as this is a common distance used in commercial SRC fields. To prevent competition and ensure plant establishment, we used a weed control mat, with borders approximately 3 m in width. The distances between plots within and among blocks were at least 180 m. We randomly assigned the different treatments within 10 blocks of three plots each. Underwood et al. (2014) suggested that associational effects should be defined as effects of frequencies of resource organism types other than that of the focal plant, separated from focal resource organism type density effects. However, because we kept genotypic variation and number of plants constant while varying sex ratios we could not separate potential effects of focal plant sex density from effects of neighbor plant sex frequencies. Due to heavy roe deer herbivory decreasing plant sizes in 2013, we fenced all plots in December 2013, and evened out plant sizes across plots by harvesting all plants using garden shears in January 2014. We recorded the severity of infection by naturally colonizing *Melampsora* spp. on plants in September 2014 using an assessment scale ranging from 0 (no infection) to 6 (most severe infection) that was based on symptoms and approximate proportion of infected leaves (c.f. Table 2 in Pei et al. 2008).

Of the 1200 plants, 140 died during the course of the experiment. By the time of infection scoring, the leaves of most neighboring plants had been in physical contact for approximately 1 month because of plant sizes and spacing. No plants in the field experiment flowered during 2014. We excluded one male clone from all analyses because its morphology and secondary metabolite content suggested that it was probably a hybrid or a member of a species other than *S. viminalis*, and three male clones and one female clone were replaced in early 2014 because they had been identified as the same genotype as other clones or incorrectly sexed.

Between 22 April 2015 and 1 May 2015, we measured phenology by recording Julian date of the first leaf of each surviving tree in all plots within one block to assess exposure time to spores. Maximum time between phenology recordings was 3 days. To assess leaf area, we measured width at the broadest point along the leaf and length of the leaves on the 30 July 2015, using a caliper. Leaf measurements were taken on mature leaves of a middle‐height branch of each plant in all three plots within the block with the highest survival. We calculated leaf area (*A*) as the form of an ellipse (*A* = *πab*), using leaf length for the long axis (*a*) and width for the short axis (*b*).

### Literature survey

To examine which sex‐biased infection is most common, we collected all studies we could find that reported plant sex differences in pathogen infection starting with lists from two older reviews (Ågren et al. 1999; Vega‐Frutis et al. 2013). We searched Web of Science (Thomson Reuters 2015) using search terms related to dioecy and pathogens, and also searched studies cited in studies that we had found initially. Because studies used various methods to quantify infection, we used vote counting rather than statistical testing. Each unique plant–pathogen species pair was given one vote regardless of the number of supporting studies, except for where evidence were mixed, in which case the species pair was given one vote for male‐biased and one vote for female‐biased infections.

To further evaluate the generality of plant sex effects in *S. viminalis* on infection by *Melampsora* spp., we searched Web of Science (Thomson Reuters 2015) using the two species names as keywords. We surveyed studies that included information on the severity of *Melampsora* spp. infection on *Salix viminalis* clones in those cases where at least two clones of each sex were examined. We noted the sex of the most severely infected clone, regardless of statistical significance. In case of a tie, we counted the number of clones of each sex in the highest severity category because most studies used ordinal scales for severity scores. We used information from a *Salix* clone archive at the Swedish University of Agricultural Sciences, Uppsala, Sweden (Moritz, unpublished data), and the National Willow Collection at Rothamsted Research, Harpenden, Great Britain (William Macalpine, personal communication) to determine the sex of clones included in the studies that we surveyed.

### Statistical analyses

We used the statistical software R 3.2.0 (R Development Core Team 2015) for all analysis. We used the *clmm* function in the *ordinal* package in R to create a cumulative link mixed model (CLMM) with the severity of infection on each plant as an ordinal response variable. We included plant sex and plot sex diversity (monosexual or mixed sex) as fixed factors, and clone, and plot nested within block because of spatial variability, as random factors. We did not include plant height as a covariate, or information on replaced clones, in the final CLMM because these two terms were insignificant. We used the *lsmeans* package in R to predict estimated values in the model for plotting and the figure was created using the *ggplot2* package. To test for interactions between genotype and neighborhood sex ratio, we used a CLMM with an interaction term between genotype and plot treatment, including block and plot as random factors, and compared it to the CLMM with additive effects only. Because 140 of the 1200 plants died during the experiment, we analyzed survival using the *glmer* function in the *lme4* package to build a generalized linear model with a binomially distributed error structure and log link, with survival as a response variable. We used plant sex and plot sex diversity (monosexual or mixed sex) as fixed factors, and included clone, and plot nested within block, as random factors. To compare phenology and leaf areas for sexes of plants within one block, we built models using the *lmer* function in the *lme4* package with plant sex as a fixed factor and plot as a random factor, and used Kenward–Roger approximations with the *Anova* function in the *car* package. Calculated leaf areas were square root‐transformed prior to testing.

Because most of the studies that we found in the literature survey had measured infection using different types of data or at different scales, we did not perform statistical testing on our literature survey findings. Instead, we relied on vote counting where for each study, the plant sex with the highest scores was given one vote. Most *S. viminalis* clones were of Swedish or British origin, and several of these were present in more than one study. Many of the studies included only a few clones of one or both sexes, or had heavily skewed sex ratios.

## Results

**Figure 2:**
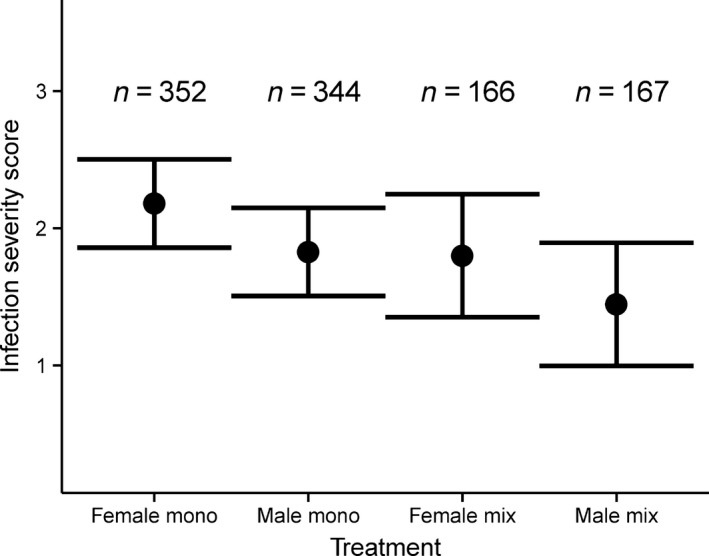
Fitted mean scores for severity of *Melampsora* infection on male and female *Salix viminalis* in monosexual and mixed sex plots ± standard errors. *n* denotes numbers of trees.

The sex of focal *Salix viminalis* plants, rather than associational plant sex effects, drove the variation in severity of infection by *Melampsora* spp. Female plants had higher scores for *Melampsora* spp. infection severity than male plants (Table 1, Fig. 2), but treatment (monosexual or mixed sex plots) did not significantly affect severity of infection (Table 1, Fig. 2), and the interaction between plant sex and treatment was not significant (*P* = 0.883). Infection severity scores ranged between 0 and 5, and fitted mean scores for treatments corresponded to between light and medium‐severe infections. Furthermore, plant survival did not differ among treatments (df = 1, *z* = 1.015, *P* = 0.310) or between sexes (df = 1, *z* = 1.346, *P* = 0.178). We found no significant interaction between genotype and treatment (df = 38, *P* = 0.143). Neither leaf area (df = 1, *t* = 0.56, *P* = 0.709) nor phenology (df = 1, *t* = 1.1, *P* = 0.471) differed between the sexes.

**Table 1:** Effects of independent factors on severity of *Melampsora* spp. infection in the cumulative link mixed model

Factor	Estimate	Standard error	z‐Value	*P*
Treatment	0.930	1.320	0.705	0.481
Plant sex	−0.864	0.317	−2.728	0.006

In total, we found female‐biases in nine of 13 plant–pathogen species pairs for which sex‐biased infections in dioecious plants have been investigated, and male‐biases in five of the species pairs (Table 2). For plant–pathogen pairs where the infected plant tissue was leaf tissue, four gave support for female‐biased and three for male‐biased infection. For species pairs where the infected tissue was floral, four had support for female‐biased and two for male‐biased infection, while two had in one study each been found not to be plant sex‐biased. Our literature survey of *Melampsora* spp.‐infected *S. viminalis* consisted of seven studies (Table 3) and vote counting supported female‐biased infection severities being more common (five of seven previous studies, excluding the present study). It should, however, be noted that most studies included more female clones, possibly influencing these results. All the studies had used mixed clone plantations with sexes mixed, making it difficult to assess associational effects of plant sex in *S. viminalis*. Furthermore, four studies in the literature survey had been performed in plantations of mixed species (Bell et al. 2001; McCracken and Dawson 2003; Pei et al. 2008; Begley et al. 2009).

**Table 2:** Reported plant sex‐biased fungal infections of dioecious plants. *Silene latifolia* was formerly known as *Silene alba* and *Microbotryum violaceum* as *Ustilago violacea*. n.d. = no difference

Plant	Pathogen	Conditions	Bias	Tissue	Study
*Arisaema triphyllum*	*Uromyces ari‐triphylly*	Field	Female	Leaves	Lovett Doust and Cavers (1982)
*Baccharis halimifolia*	Unidentified	Field	Male	Leaves	Caño et al. (2013, 2014)
*Buchloe dactyloides*	*Puccina kansensis*	Greenhouse	Male	Leaves	Quinn (1991)
*Bouteloua dactyloides*	*Salmacisia buchloëana*	Laboratory	Female	Flowers	Chandra and Huff (2014)
*Cannabis sativa*	*Botrytis cinerea*	Laboratory	Female	Flowers	McPartland (1996)
*Cannabis sativa*	*Dendrophoma marconii*	Field	Male	Leaves	Gikalov (1935)
*Juniperus communis*	Unidentified	Field	Female	Roots	Ward (2007)
*Pistacia vera*	*Alternaria alternate*	Laboratory	Female	Leaves	Ash and Lanoiselet (2001)
*Populus cathayana*	*Melampsora larici‐populina*	Laboratory	Female	Leaves	Zhang et al. (2009)
*Rubus chamaemorus*	Unidentified	Field	Male	Leaves	Ågren (1987)
*Salix viminalis*	*Melampsora* spp.	Field	Female	Leaves	Åhman (1997); Present study
*Silene dioica*	*Microbotryum violaceum*	Field	n.d.	Flowers	Baker (1947)
*Silene dioica*	*Microbotryum violaceum*	Field	Male	Flowers	Hassan and MacDonald (1971)
*Silene dioica*	*Microbotryum violaceum*	Field	Female	Flowers	Lee (1981)
*Silene latifolia*	*Microbotyrum violaceum*	Field	n.d.	Flowers	Alexander (1989, 1990)
*Silene latifolia*	*Microbotryum violaceum*	Laboratory, field	Female	Flowers	Alexander and Antonovics (1988); Alexander (1990); Alexander and Maltby (1990); Shykoff et al. (1996); Kaltz and Shykoff (2001)
*Silene latifolia*	*Microbotryum violaceum*	Field	Male	Flowers	Thrall and Jarosz (1994); Alexander and Antonovics (1995); Biere and Antonovics (1996)

**Table 3:** Studies on severity of *Melampsora* spp. infection on male and female clones of *Salix viminalis*

Study	Settings	Sexual diversity	Assessment	Data	Females/males	Most severely infected
Lascoux et al. (1996)	Lab, inoculation	Mix (growth chambers)	Uredina	Numeric	4/4	Females
Åhman (1997)	Field	Mix	Severity	Ordinal	413/115	Females
Bell et al. (2001)	Field	Mix and monocultures	Severity	Qualitative	3/8	Females
McCracken and Dawson (2003)	Field	Mix and monocultures	Severity	Ordinal	3/7	Males
Pei et al. (2004)	Lab, inoculation	Separated leaves	Severity	Ordinal	23/15	Females
Pei et al. (2008)	Field	Mix	Severity	Ordinal	2/3	Males
Begley et al. (2009)	Field	Mix, clonal monocultures	Severity	Ordinal	3/2	Females
Present study	Field	Mix, sexual monocultures	Severity	Ordinal	20/19	Females

## Discussion

Here, we show that although plant sex can affect the severity with which an individual plant is infected with fungal disease, there were no strong associational plant sex effects. Our literature survey indicates that female‐biased fungal pathogen infections are also more common in other plant–pathogen species pairs. While our field experiment reveals that female plants are more severely infected, there were no strong neighborhood sex ratio effects, regardless of the sex and genotype of the focal plant.

Contrary to our prediction that a balanced sex ratio would reduce the severity of infection in our experiment, as higher genotypic or species diversity in various systems reduce disease (e.g. Zhu et al. 2000), we found neither effects of neighborhood sex ratio nor interactive effects between the focal plant genotype or sex and neighborhood sex. To assess the generality of plant sex and associational plant sex effects for *S. viminalis*, we included 20 clones per plot in our study, whereas natural populations often have low genotypic diversity, and the most common practice in SRC plantations is planting only one or two clones. Therefore, genotypic diversity in our experiment was higher than that of most natural and agricultural stands, which could have led to lower overall infection severity. Mixing clones of *S. viminalis* in plantations reduces the severity of fungal disease compared to that in monocultures (Begley et al. 2009), probably because greater genetic variation reduces infection, while genotypes present in a stand of low genotypic diversity will strongly affect infection. Indeed, it has been proposed that taking sex into account when selecting willow clones for agriculture is unnecessary because large interclonal variations in resistance will outweigh any differences between sexes (Åhman 1997). If there are no associational sex effects on infection severity, plant sex may have only minor effects on infection relative to genotype effects in low‐diversity stands. A study manipulating both genetic or genotypic diversities and sex ratios would assess whether the lack of associational plant sex effects is true irrespective of intraspecific plant diversity.

Although life history trade‐offs suggest that female plants should be better defended against pathogens (Vega‐Frutis et al. 2013), female‐biased fungal infections appear to be more common; for 12 plant species, we found empirical evidence for female‐biased infections in nine and male‐biased infections in six. Three plant species showed sex‐biased infections in both directions, indicating that sex‐biases may be influenced by other factors than plant sex, for example, the type of tissues that are infected, plant–pathogen pair, or disease vectors.

Possible main explanations for susceptibility differences between male and female plants to leaf fungal pathogens are intersexual variation in exposure (i.e., the target area for spores or time of exposure), biochemical defences, or structural defences such as trichomes (Ågren et al. 1999). Furthermore, intersexual differences in phenology may lead to different exposure because of different cumulative infection risks over time. Larger leaf area in females have been reported for other *Salix* species (Ueno and Seiwa 2003) although the opposite is generally true for dioecious plants (Cornelissen and Stiling 2005) including at least one other *Salix* sp. (Dawson and Bliss 1989). The severity of *Melampsora* spp. infection is closely correlated with leaf area in *S. viminalis* (Toome et al. 2009), but we did not find differences in calculated leaf areas or phenology, suggesting that intersexual differences in exposure unlikely affected infection. We did not quantify leaf chemical defences in our experiment. Female plants often contain higher concentrations of secondary metabolites (Ågren et al. 1999) and it is possible that leaf chemistry could have resulted in the sex‐biased infection severity in our experiment. Previous studies have, however, not detected any relationship between secondary compound content and *Melampsora* spp. infection in *Salix myrsinifolia* (Hakulinen 1998; Hakulinen and Julkunen‐Tiitto 2000). Finally, *Salix viminalis* leaves have short trichomes and we did not measure trichome density in our experiment. Further research clearly needs to investigate traits underlying sex‐bias in the infection of fungal disease in dioecious plants and their relative importance.

We have shown here that for *S. viminalis*, differences in susceptibility are not modulated by neighborhood plant sex ratio in an even‐sex mixture compared to monocultures. Our study demonstrates that regardless of sex or genotype, the severity of infection on individual *S. viminalis* is not significantly affected by the sex of neighboring conspecifics at these sex ratios. We also found that female *S. viminalis* plants experience more severe *Melampsora* spp. infections both in field experiment and literature survey. However, because sex ratios were often skewed, the literature survey on *S. viminalis* and *Melampsora* should be interpreted with caution. Our survey of sex‐biased fungal infections in dioecious plants reported in previous studies shows that most infections were female‐biased. Our study suggests that plants in monosexual natural stands and plantations may not suffer infections that are more severe than those whose neighborhoods have equal sex ratios, at least at this level of genotypic diversity. The mechanisms behind plant sex‐biased fungal pathogen infections in dioecious plants, however, remain an unresolved issue, and we suggest that further studies investigate plant and pathogen traits that may cause sex‐biased infections.

## Data Accessibility

Field experiment data that was used in infection severity and survival models, on leaf measurements and on phenology, and R script was used for statistical analyses: Data available from the Dryad Digital Repository: http://dx.doi.org/10.5061/dryad.dd687.

## Conflict of Interest

None declared.

## Supporting information


**Appendix S1.** *Salix viminalis* clones and their mean *Melampsora* spp. infection severities in the field study, listed by sex and name.

**Data S1.** R script for field experiment.
