# Supplementary material for: Female Salix viminalis are more severely infected by Melampsora spp. but neither sex experiences associational effects
Source: Ecol Evol. 2016 Jan 25;6(4):1154–62. doi: 10.1002/ece3.1923 (PMC4725332; doi:10.1002/ece3.1923)
Supplement: Supplementary file 1 — Appendix S1. Salix viminalis clones and their mean Melampsora spp. infection severities in the field study, listed by sex and name. [file ECE3-6-1154-s001.docx]

Appendix 1. Salix viminalis clones and their mean *Melampsora spp.* infection severities in the field study, listed by sex and name. Phenology max and min denote extremes for first leaves of plants. Clone 81092 was excluded from all analyses. Phenology and leaf measures represent means of one block in the field experiment.

| Sex | Clone | Mean infection severity score (n=21-30) | Phenology max 2015 (Julian days, n=2-4)) | Phenology min 2015 (Julian days, n=2-4)) | Mean leaf width 30^th^ July 2015, (n=2-4) | Mean leaf length 30^th^ July 2015, (n=2-4) |
| --- | --- | --- | --- | --- | --- | --- |
| Female | 78003 | 2.21 | 121 | 118 | 1.50 | 14.45 |
| Female | 78021 | 2.14 | 118 | 114 | 1.88 | 14.78 |
| Female | 78090 | 2.26 | 117 | 114 | 1.63 | 14.70 |
| Female | 78091 | 2.32 | 114 | 114 | 1.35 | 10.30 |
| Female | 78112 | 2.29 | 121 | 112 | 1.45 | 14.15 |
| Female | 78120 | 2.25 | 117 | 117 | 1.30 | 12.45 |
| Female | 78183 | 2.31 | 117 | 117 | 1.20 | 14.35 |
| Female | 78198 | 2.63 | 118 | 118 | 1.65 | 12.65 |
| Female | 811113 | 2.96 | 121 | 114 | 1.60 | 11.50 |
| Female | 811126 | 2.39 | 118 | 117 | 1.55 | 16.25 |
| Female | 811208 | 1.82 | 121 | 114 | 1.55 | 14.05 |
| Female | 811210 | 1.69 | 117 | 114 | 1.55 | 13.45 |
| Female | 812518 | 1.81 | 117 | 117 | 1.45 | 12.33 |
| Female | 814001 | 2.21 | 117 | 114 | 1.43 | 14.43 |
| Female | 814009 | 1.52 | 117 | 114 | 1.53 | 13.97 |
| Female | Christina (821629) | 2.20 | 117 | 117 | 1.80 | 15.80 |
| Female | Eva (810217) | 2.14 | 121 | 117 | 1.93 | 18.77 |
| Female | Hanna (821624) | 2.04 | 117 | 114 | 2.15 | 18.65 |
| Female | Jorunn | 1.59 | 118 | 117 | 1.63 | 14.00 |
| Female | Lisa (811110) | 1.64 | 117 | 117 | 1.68 | 13.38 |
| Male | 77683 | 1.86 | 121 | 118 | 2.25 | 19.40 |
| Male | 78101 | 2.00 | 112 | 112 | 1.90 | 16.05 |
| Male | 78118 | 2.04 | 117 | 117 | 1.63 | 16.00 |
| Male | 81092 | 1.72 | 117 | 117 | 2.00 | 19.30 |
| Male | 82060 | 2.61 | 117 | 114 | 1.50 | 16.95 |
| Male | 820367 | 1.91 | 114 | 112 | 1.38 | 12.83 |
| Male | 820381 | 2.21 | 118 | 117 | 1.58 | 13.53 |
| Male | 821754 | 1.92 | 121 | 117 | 1.27 | 12.20 |
| Male | 830203 | 1.52 | 121 | 114 | 1.63 | 14.73 |
| Male | 831905 | 1.61 | 118 | 117 | 2.05 | 17.20 |
| Male | Anki | 1.87 | 118 | 117 | 1.25 | 13.70 |
| Male | Gustaf (832803) | 2.07 | 118 | 114 | 1.50 | 14.10 |
| Male | H 103 | 2.07 | 117 | 117 | 1.45 | 12.35 |
| Male | Jorr (880013) | 1.60 | 117 | 117 | 1.55 | 11.45 |
| Male | Marie (832501) | 2.47 | 117 | 117 | 1.40 | 15.40 |
| Male | NL 420 | 1.46 | 117 | 114 | 1.53 | 10.88 |
| Male | Orm | 1.83 | 117 | 117 | 1.67 | 13.73 |
| Male | Rapp | 2.08 | 117 | 117 | 1.65 | 13.18 |
| Male | Tofta 13 | 1.83 | 117 | 117 | 1.70 | 14.30 |
| Male | Ulv | 2.14 | 117 | 117 | 1.40 | 11.60 |
